# Correction: Distinct expression profile reveals glia involvement in the trigeminal system attributing to post‑traumatic headache

**DOI:** 10.1186/s10194-025-01949-w

**Published:** 2025-01-14

**Authors:** Gurueswar Nagarajan, Yumin Zhang

**Affiliations:** 1 Henry M. Jackson Foundation for the Advancement of Military Medicine, 6720A Rockledge Dr, Bethesda, MD 20817 USA; 2 Department of Anatomy, Physiology and Genetics, Uniformed Services University of the Health Sciences, 4301 Jones Bridge Road, Bethesda, MD 20814 USA

**Correction: J Headache Pain 25, 203 (2024)**

10.1186/s10194-024-01897-x

In this article, an incorrect Data availability statement was published that read ‘No datasets were generated or analysed during the current study’.

The correct statement appears below:

The underlying data and computational scripts employed in this study will be shared upon request. Data used for meta-analysis can be found in Yang et al., 2022 (https://ars.els-cdn.com/content/image/1-s2.0-S0896627322002288-mmc5.xlsx and https://ars.els-cdn.com/content/image/1-s2.0-S0896627322002288-mmc13.xlsx) and data used for comparison of injury models can be found in Nguyen et al., 2019 (10.7554/eLife.49679.019).

The original article has been updated.

