# Supplementary figures and images for: Distinct expression profile reveals glia involvement in the trigeminal system attributing to post-traumatic headache
Source: J Headache Pain. 2024 Nov 22;25(1):203. doi: 10.1186/s10194-024-01897-x (PMC11585153; doi:10.1186/s10194-024-01897-x)

# Righting Reflex following Injury

(Measure of unconscious state)

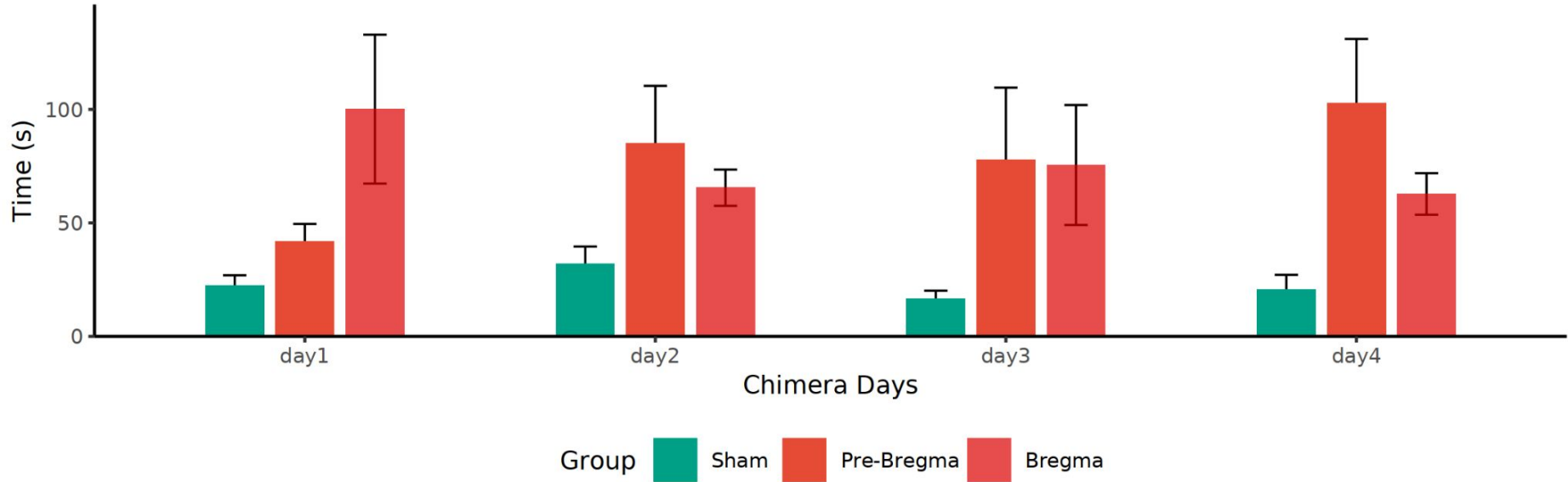

Supplement: Supplementary file 1 — Additional file 1: Righting reflex in each of four mTBI days (n=6/group). [file 10194_2024_1897_MOESM1_ESM.pdf]

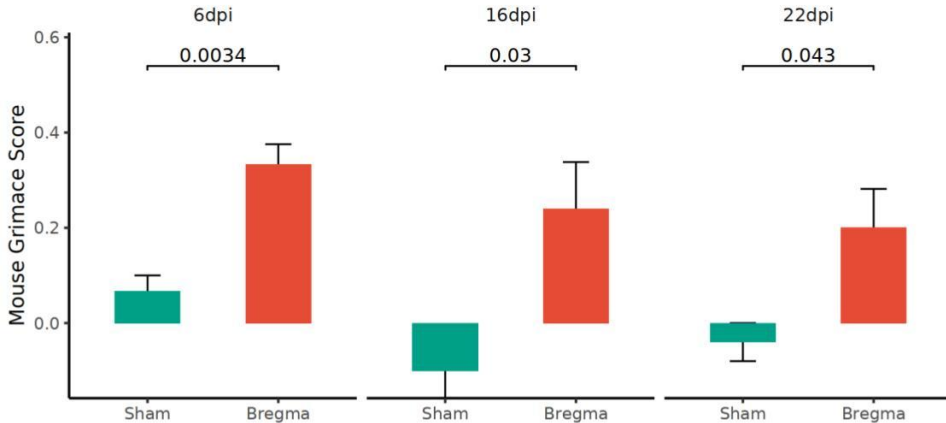

Supplement: Supplementary file 2 — Additional file 2: Bar graphs representing grimace at 6, 16- and 22-days post mTBI (dpi) in bregma position (n=4-5/group). [file 10194_2024_1897_MOESM2_ESM.pdf]

# GSEA - Brainstem Astrocyte Unique Gene Sets in the Sp5C

Astrocyte specific Gene sets

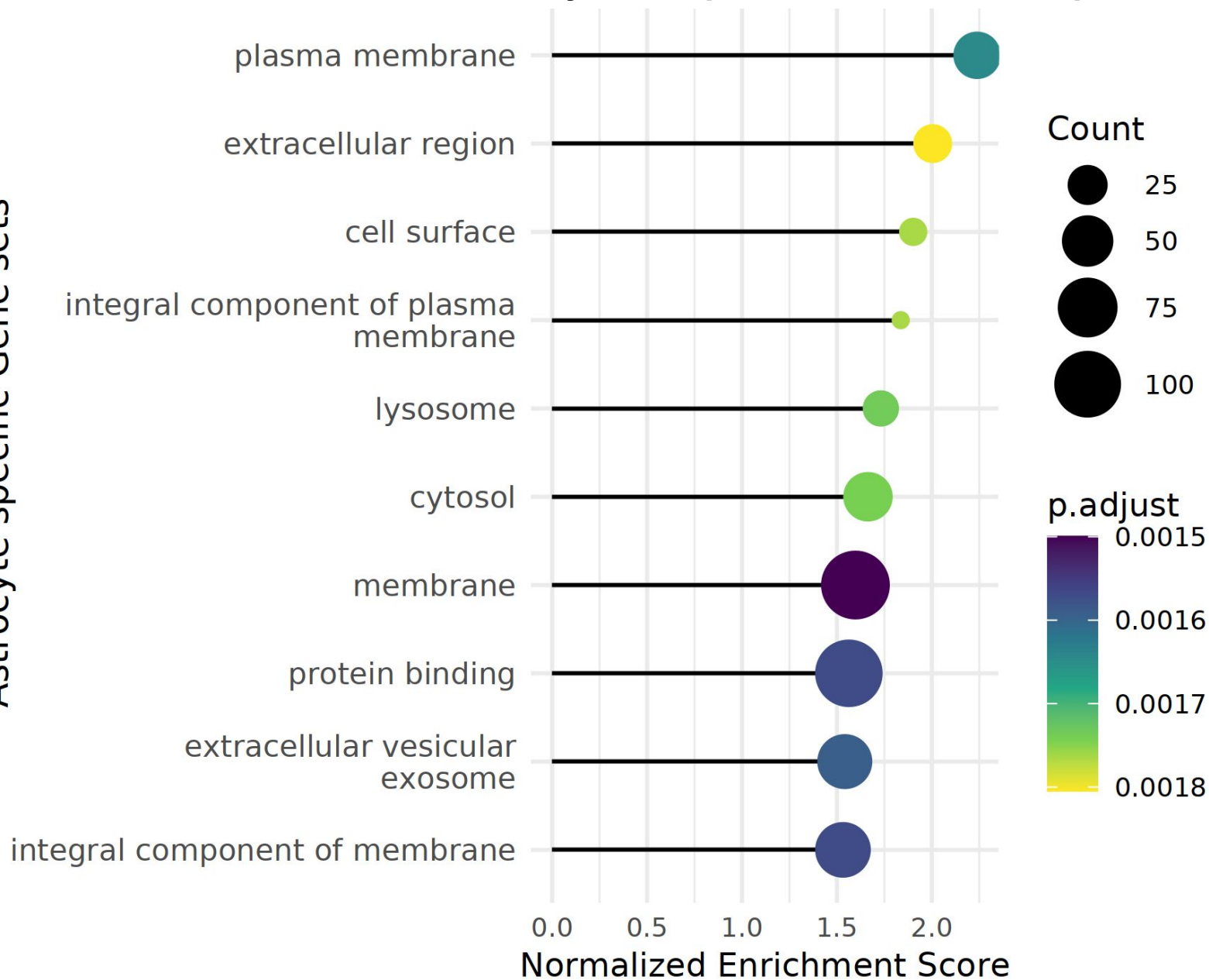

Supplement: Supplementary file 4 — Additional file 4: Dot plot representing astrocyte specific enriched gene sets in the Sp5C following mTBI. [file 10194_2024_1897_MOESM4_ESM.pdf]

**Iba1**

**CD68**

**Merged**

**Sp5C**

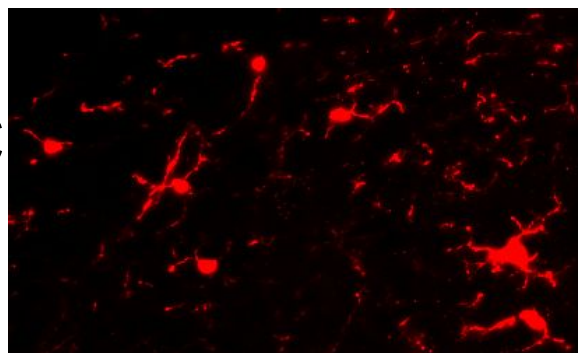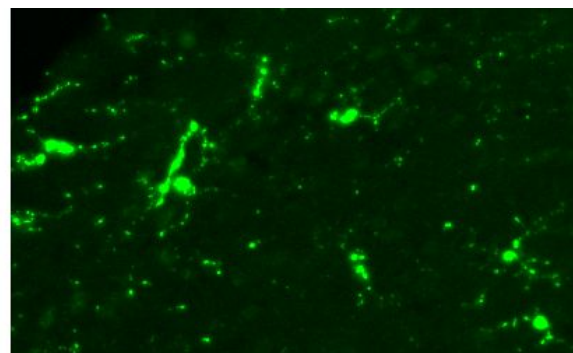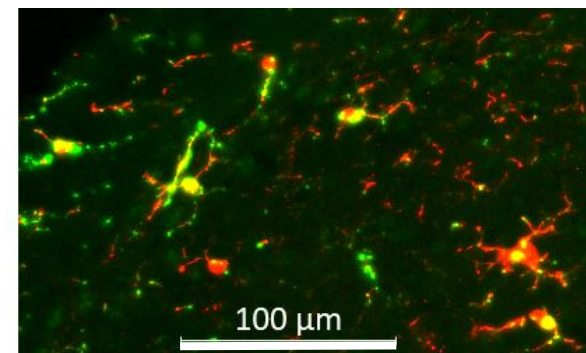

**MdV**

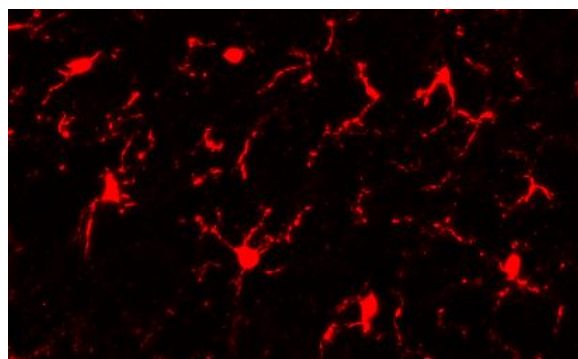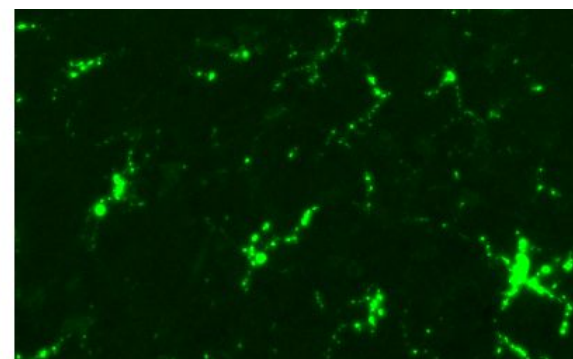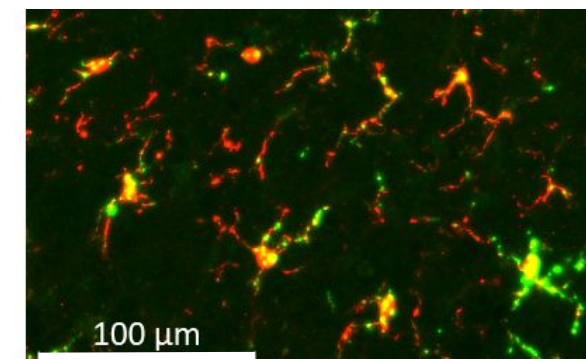

**SC**

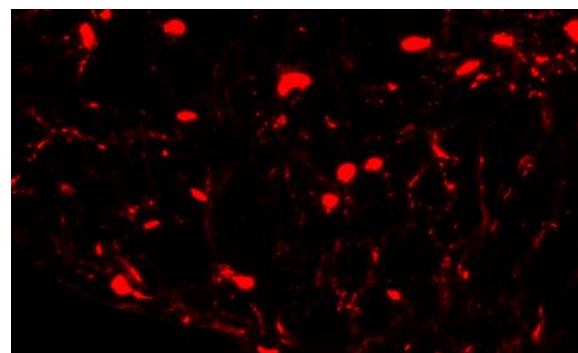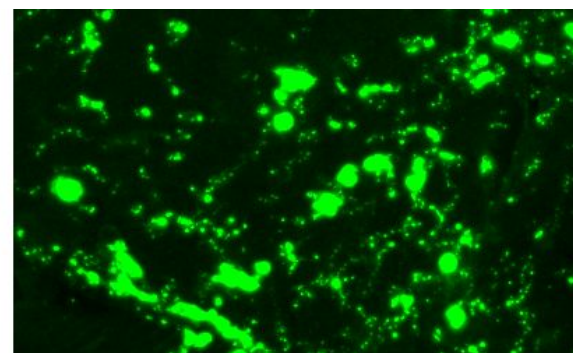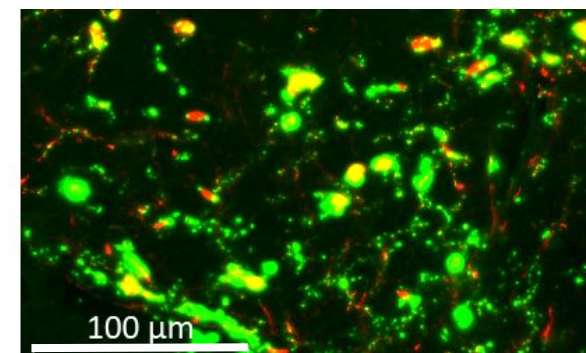

**Gr**

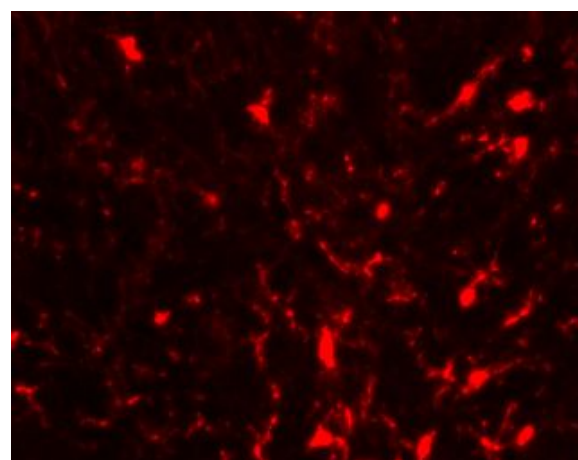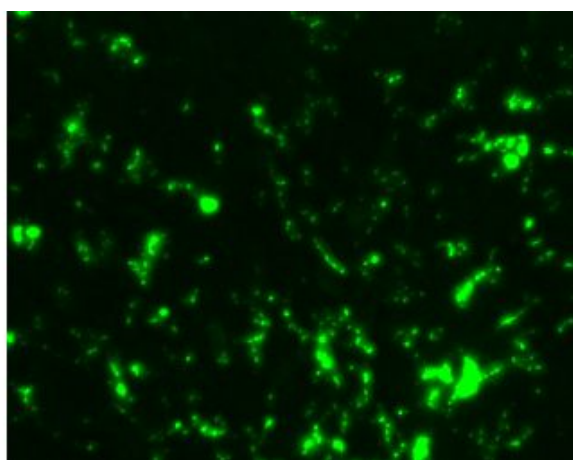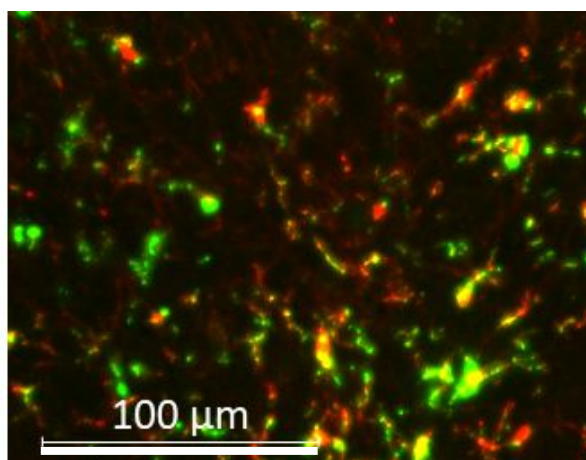

Supplement: Supplementary file 5 — Additional file 5: Distinct expression pattern of CD68+, Iba1+ cells in the brainstem. Fiber tracts, specifically spinocerebellar tract (sc), show a distinct morphological profile compared to the microglia in the gray matter (Sp5C, Gr, Mdv). Gr - gracile nucleus; Mdv - Medullary reticular nucleus. [file 10194_2024_1897_MOESM5_ESM.pdf]

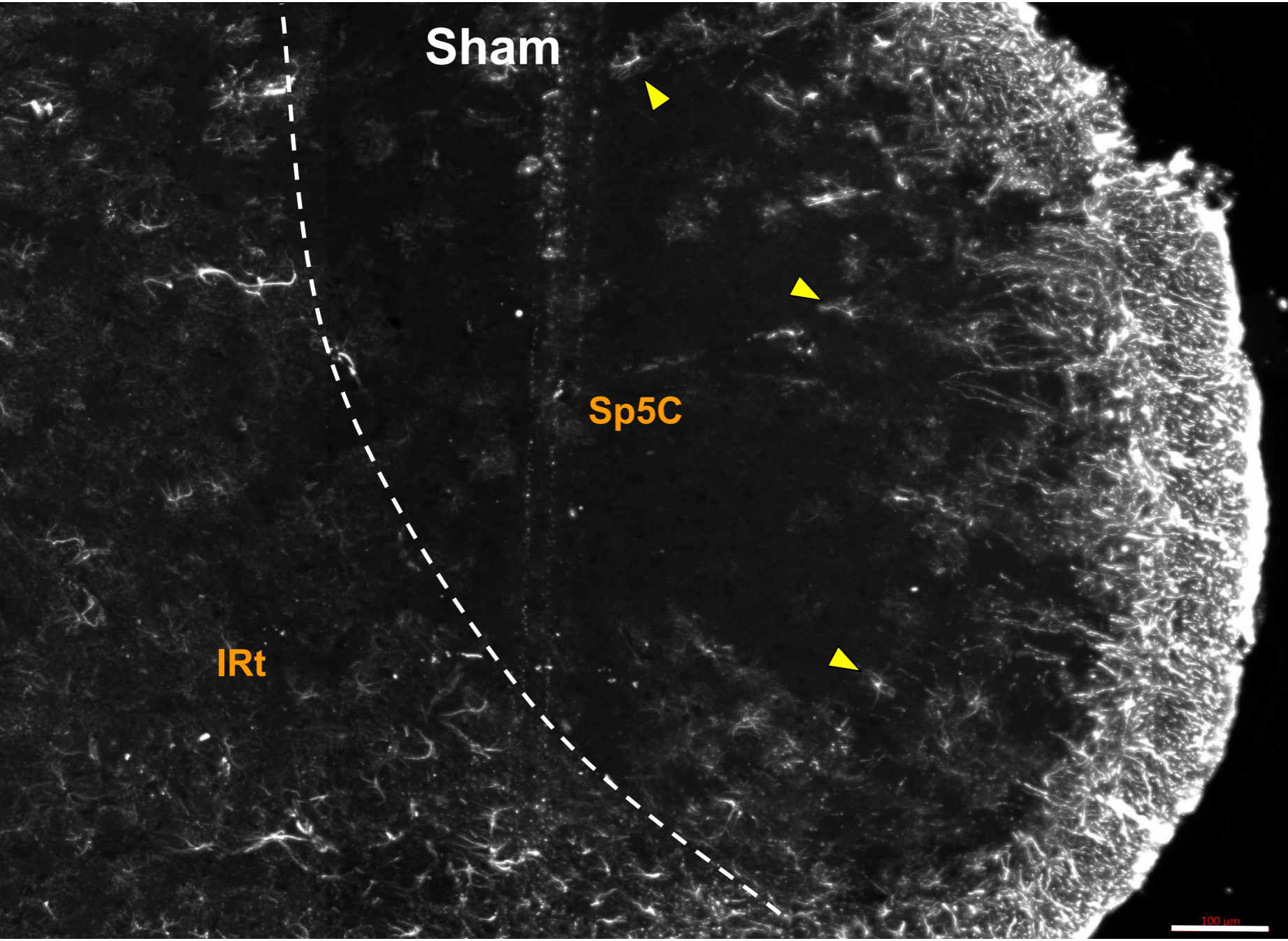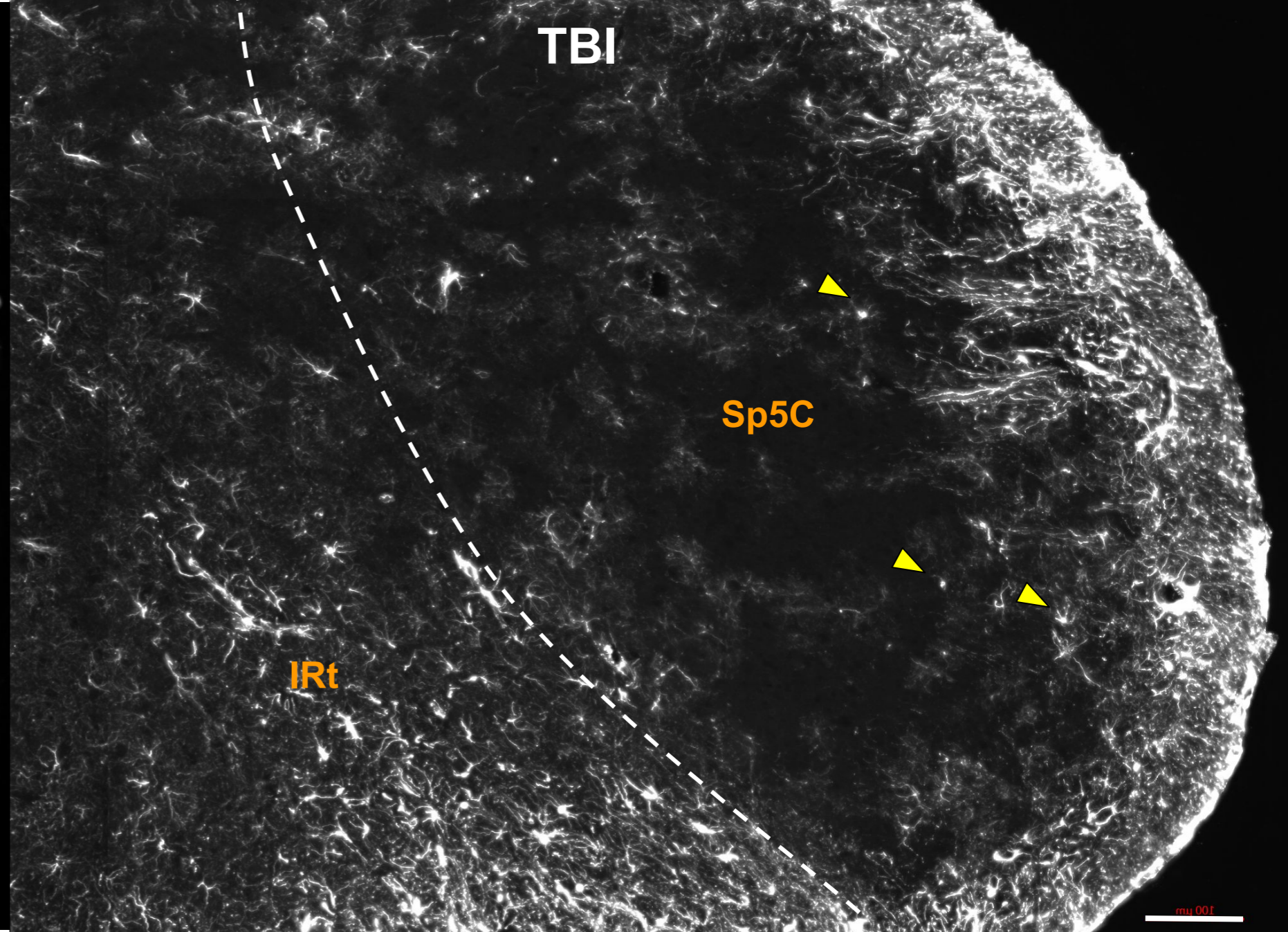

Supplement: Supplementary file 6 — Additional file 6: Increased presence of GFAP+ fibrous astrocytes in the Sp5C compared to the paratrigeminal regions. Rare appearance of protoplasmic astrocytes in the Sp5C and prominent protoplasmic appears in the reticular formation in both Sham and mTBI. IRt - Intermediate reticular nucleus. [file 10194_2024_1897_MOESM6_ESM.pdf]

**Sham**

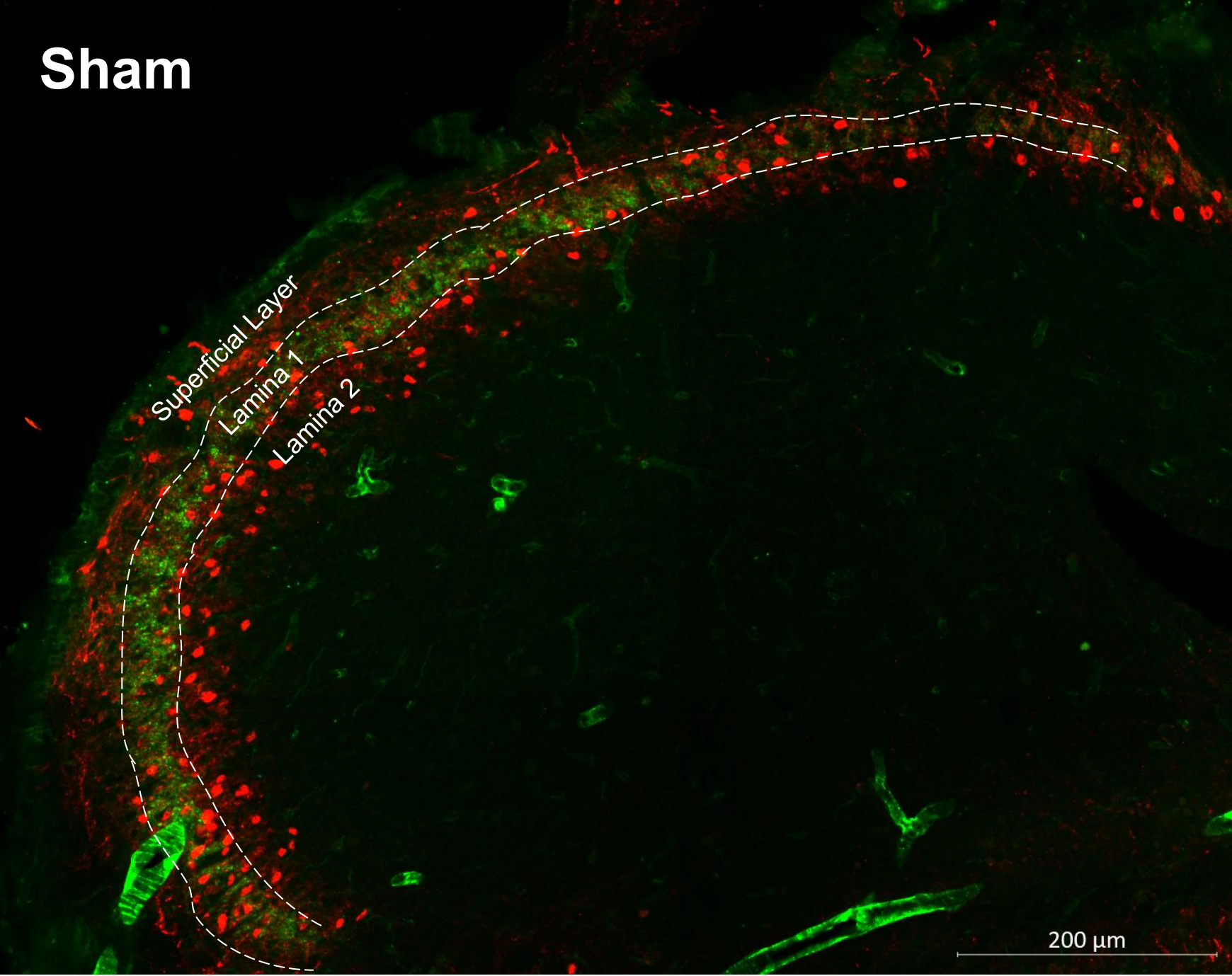

**mTBI**

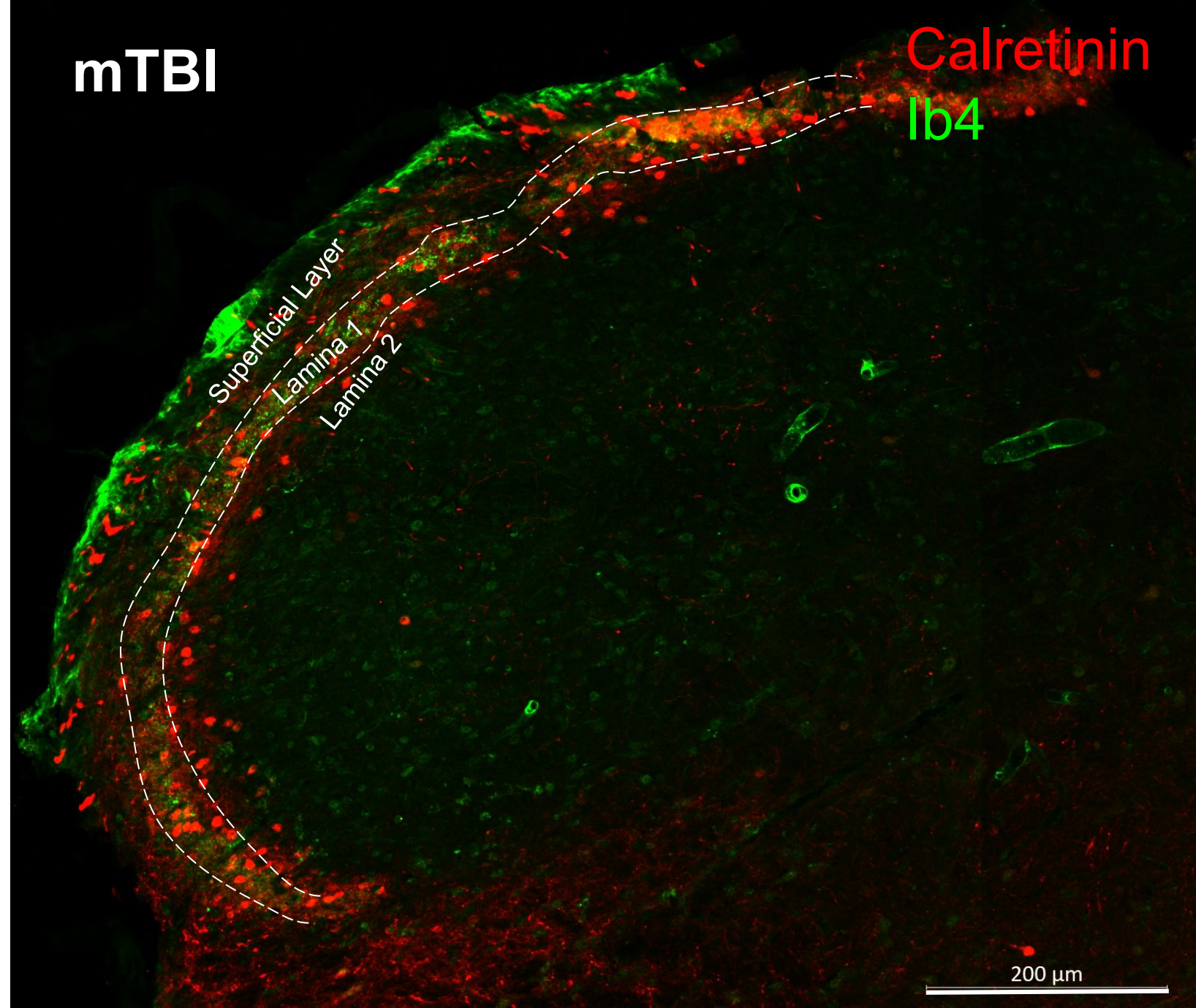

Supplement: Supplementary file 7 — Additional file 7: Representative images from sham and mTBI mice showing calretinin-ir neurons in the lamina I of Sp5C. Dotted white lines represent the Ib4+ staining. Atlas plate at the level of ~ -8.3 from bregma. Scale bar = 200 µm. [file 10194_2024_1897_MOESM7_ESM.pdf]
